# Glue and Ethanol Mixture for Aneurysm Endovascular Treatment: Animal Lab Study, Imaging, and Histopathological Findings

**DOI:** 10.3390/jcm13237222

**Published:** 2024-11-28

**Authors:** Massimo Muto, Giuseppe Leone, Flavio Giordano, Gianluigi Guarnieri, Antonio Di Donna, Vincenzo Andreone, Eva Di Maro, Alessandro Poli, Niccolò Fonti, Ferdinando Caranci, Mario Muto

**Affiliations:** 1 “Dipartimento delle Tecnologie Avanzate Diagnostico-Terapeutiche e dei Servizi Sanitari”—UOC Neuroradiologia, Azienda Ospedaliera di Rilievo Nazionale “Antonio Cardarelli”, 80131 Napoli, Italy; massimo.muto@yahoo.it (M.M.); g.leonemd@gmail.com (G.L.); flavio.giordano88@gmail.com (F.G.); gianluigiguarnieri@hotmail.it (G.G.); adidonna11@gmail.com (A.D.D.); mutomar2@gmail.com (M.M.); 2 “Dipartimento di Emergenza e Accettazione”—UOC Neurologia e Stroke Unit, Azienda Ospedaliera di Rilievo Nazionale “Antonio Cardarelli”, 80131 Napoli, Italy; andreone2@gmail.com; 3 Centro di Biotecnologie, Azienda Ospedaliera di Rilievo Nazionale “Antonio Cardarelli”, 80131 Napoli, Italy; eva.dimaro@aocardarelli.it; 4 Department of Veterinary Sciences, University of Pisa, 56124 Pisa, Italy; niccolo.fonti@phd.unipi.it; 5 Department of Precision Medicine, Università degli Studi della Campania “Luigi Vanvitelli”, 80138 Naples, Italy; ferdinando.caranci@unicampania.it

**Keywords:** glue, angionecrosis, vascular malformations treatment, endovascular embolization, experimentally induced aneurysm, N-butyl cyanoacrylate

## Abstract

**Background:** This study aims to investigate the degree of penetration, permanence of occlusion, and vascular changes induced by a newly modified mixture of n-butyl cyanoacrylate (Glubran 2^®^), ethanol, and Lipidol^®^ (GEL) in the endovascular treatment of experimental aneurysms induced in swine. **Methods:** Bilateral pouch aneurysms were created in the wall of the internal carotid artery in eighteen pigs. The sixteen aneurysms were treated with a new mixture of GEL with different component proportions. Angiograms were obtained at the time of treatment and at 1, 4, and 16 weeks after treatment. According to the scheduled experimental design, subjects were sacrificed at the time of treatment and 7, 30, and 90 days after the embolization of experimentally induced aneurysms. The internal carotid artery and aneurysms were removed en bloc and sampled for histopathologic study. **Results:** The mixture induced complete and stable occlusion without recanalization in all experimentally induced aneurysms throughout the study period. Histopathological studies showed focal angionecrosis and acute inflammatory reactions from 7 dpi, followed by chronic inflammation and vessel wall thickening. **Conclusions:** The GEL mixture may be useful in future clinical applications for the embolization of arteriovenous malformations, the control of acute bleeding, and the isolation of aneurysms due to its very short polymerization time and minimal adhesion to the microcatheter.

## 1. Introduction

Medical glue embolization, first used in 1975, is permanent and instantaneous, but it can be toxic if infused into the intravascular lumen, making it suitable for specialized centers [1]. Intermediate-length cyanoacrylate adhesives like n-butyl 2-cyanoacrylate (NBCA) were the first product to be broadly used in medicine for closing cutaneous wounds and are now mostly utilized to permanently occlude vascular anomalies, such as brain and spinal vascular malformations [2], as well as in portal vein embolization [3], pre-operative renal tumor embolization, the embolization of endoleaks through endoprostheses, and embolization for bleeding control [4].

Histoacryl^®^ (B/Braun, Tuttlingen, Germany) or N-butyl-2-cyanoacrylate glue is the most commonly used. It has replaced isobutyl cyanoacrylate and is also marketed under the name Trufill^®^ (Cordis, Miami Lakes, FL, USA). The lack of formal acknowledgment for Histoacryl^®^’s off-label use in endovascular therapy has garnered attention in recent years, giving rise to serious legal issues. Because of this, a brand-new acrylic glue called Glubran 2^®^ (GEM SRL, Viareggio, Italy) was introduced to the market in 2000, wherein N-butyl-2-cyanoacrylate and metacryloxysulpholane (MS) are mixed to create a more flexible polymer with a milder exothermic reaction (45 °C) that causes less inflammation and histotoxicity [5,6,7]. Metacryloxysulpholane also gives surgical Glubran 2^®^ a significant anti-inflammatory effect. The addition of MS to NBCA yielded a glue complying with European regulations, hence receiving CE certification for internal and endovascular use. Lipiodol^®^ is usually added to NBCA for visibility under fluoroscopic control [8,9,10], and the polymerization time can be modulated by changing the Lipiodol^®^-to-NBCA ratio [11,12,13].

Further studies showed that ethanol addition can accelerate the polymerization time [14,15,16]. Kawai et al. (2012) observed that adding ethanol to the combination of NBCA and Lipiodol^®^ has the consequence of changing the structure of NBCA polymerization [15]. They investigated the properties of an NLE (NBCA + Lipiodol^®^ + ethanol) mixture with increasing ratios of aneurysm packing in a swine model and discovered that high ethanol ratios caused solid-like qualities of the mixture with strong occlusive ability and little adherence to the microcatheter. Over the last few years, new embolic agents have also been developed with improved handling characteristics and penetration in artero-venous malformations (AVMs) [17]. Among these non-adhesive liquid embolic materials, so-called gelling solutions, some are now largely used for endovascular treatments [18]. Gelling solutions are polymers in solvents that solidify in situ when water replaces the solvent. The first gelling solution, described by Taki in 1990, was made by an ethylene-vinyl-alcohol copolymer (EVOH) in a dimethyl-sulphoxide (DMSO) suspension [19]. These non-adhesive liquid embolic materials, easily injected through a microcatheter, dissolve in aqueous solutions like blood, precipitating a spongy polymer cast [20,21], but endovascular DMSO shows an angio- and neurotoxic effect [22,23,24]. Still, the inflammatory reaction in the vessel wall is less pronounced than with cyanoacrylates and there is no inflammatory reaction in the surrounding interstitium [25].

Although the endovascular treatment of AVMs with embolic agents, both adhesive and non-adhesive, is nowadays largely used as a standard of care in neurovascular interventions [26], the same treatment cannot be applied for cerebral aneurysms as the long polymerization time of these embolic agents leads to a high risk of uncontrolled migration and thus damage to healthy vessels and tissues. None of the studies available in the literature investigated the use of the mixture Glubran 2^®^ (n-butyl-cyanoacrylate + metacryloxysulfolane NBCA-MS) associated with ethanol and Lipiodol^®^ as a new embolic agent for aneurysm endovascular treatment. This study aimed to investigate the degree of penetration, permanence of occlusion, and vascular changes induced by a modified mixture of an n-butyl cyanoacrylate (Glubran 2^®^), ethanol, and Lipidol^®^ (Guerbet Laboratories, Roissy, France) mixture (GEL) in experimental aneurysms induced in swine. The primary endpoint was to assess the efficacy and safety of the embolization treatment with the GEL. The secondary endpoint was to characterize, by histopathological analysis, the tissue response to this mixture.

## 2. Materials and Methods

### 2.1. Experimental Design

In this preclinical study, tissue samples from nine healthy swine, six females and three males, weighing 50–70 kg were used. The experiments were performed at the Biotechnologies Research Centre of A.O.R.N. “Antonio Cardarelli” Hospital. Permission to conduct this experimental study was granted before starting the study by the Institutional Committee on Research-Animal Care and the Italian Ministry of Health (Authorization n. 139/2019-PR dated the 18 February 2019), in according with Italian Laws DL.gs 26/2014 and Directive 2010/63/EU on the protection of animals used for scientific purposes. For each swine, two aneurysmal defects were created to achieve a total of eighteen. Before surgery, each animal was administered zoletil 50/50 + propofol + ketamine + sevorane (0.5 mL kg + 6 mg/kg + 10 mg/kg + sev.2%) and butorphanol as pre-operative analgesia. General anesthesia was induced with isoflurane gas via tracheal intubation. During the procedures, cardiac and pulmonary parameters were continuously monitored.

### 2.2. Creation of Aneurysm

Each swine underwent surgical exposure of the left and right carotid arteries and jugular veins to form two aneurysms. To generate an aneurysm model with carotid artery interruption, a 3 cm section of the jugular vein was removed first. The resected vein was then sutured to the ipsilateral carotid artery. Every swine underwent the surgical creation of the aneurysms, two aneurysms for each swine to achieve a total of eighteen aneurysms. Subsequently, femoral access was performed to place a carrier catheter in one of the two carotid axes with the use of a double microcatheter. The first was used for the injection of the GEL mixture, while the second microcatheter had a distal balloon that allowed the blocking of the flow at the aneurysm sac, thus preventing the uncontrolled migration of embolizing material from the sac itself. The animals were placed in anti-aggregative therapy to avoid spontaneous thrombosis of the created bag. Tramadol was administered at a dosage of 10 mg/kg orally via drinking water for the initial three days, while antibiotic therapy was maintained for seven days. Daily assessments were conducted on animals to identify any signs of adverse effects.

### 2.3. Gel Used for Embolization

For embolization, a GEL mixture composed of Glubran 2^®^ NBCA + MS (GEM Srl; Viareggio (LU), Italy) was used, which was withdrawn through a 21-gauge needle and mixed with the contrast agent, Lipiodol^®^ (Guerbet Laboratories, Roissy, France) and ethanol (99.9%, FUSO Pharmaceutical Industries, Ltd., Osaka, Japan). Glubran 2^®^ was mixed with ethanol and Lipiodol^®^ in the ratio 2:0.5:1 (57% Glubran 2^®^, 14% EtOH, 28%Lipiodol^®^). This ratio was used because it yields a mixture that polymerizes in longer times, allowing for the controlled filling of the entire aneurysm. It was prepared by first mixing ethanol and Lipiodol^®^ in a vial, which was shaken by hand for 1 min, and then adding the NBCA + MS and shaking it all for three minutes. The amount of the injected GEL mixture was variable to the size of the created aneurysm. We measured the volume of the aneurysm and estimated the total volume of GEL required.

### 2.4. Endovascular Procedure

In each swine, packing of the aneurysm was attempted 3–4 h after the creation of the aneurysms. The embolization procedure was performed on the swine at an angiography facility (GE Healthcare, Chicago, IL, USA). Heparin (50 U/kg) was infused intravenously prior to the operation and embolization to maintain an activation clotting time of 200 s or higher (normal range < 120 s). After setting the tube angle for the best view of the lateral side of the aneurysm, digital subtraction angiography, test injection, and the interventional procedure were carried out. Carotid arteriography was conducted via a guiding catheter (Envoy 5 Fr or 6 Fr, Cerenovous, Miami, FL, USA) placed in the target vessel within which a 0.017 inch microcatheter (SL10, Stryker, Kalamazoo, MI, USA) was advanced to the aneurysm neck and inside the sac, using a 0.014-inch micro-guide wire (Transend EX; Boston Scientific, Natick, MA, USA). Afterward, a 4,35 Fr Remodeling Balloon catheter (Copernic, Balt, Montmorency, France) was inserted into the carotid artery and inflated to 6 atmospheres at the aneurysm’s neck. The mean diameter of the carotid arteries was 5.2 mm (range 4.4–5.9 mm); thus, a 6 mm balloon catheter was utilized. The gluing microcatheter was purged with saline to remove any residual contrast material and was subsequently primed with 5% glucose, using a volume that corresponded to the catheter’s dead space (approximately 1.2 mL) to prevent the polymerization of NBCA in the microcatheter. After a new control of activated clotting time of >200 s, the carotid artery was occluded by balloon inflation. The mixture was gradually injected in small increments from the distal end of the aneurysm sac using a 3 mL syringe to prevent regurgitation in the microcatheter. Following the initial injection of approximately 1–1.3 mL of GEL, a test injection was conducted. The mixture was administered gradually via the microcatheter while under fluoroscopic guidance. After packing was completed, the balloon catheter was deflated 5 min later, and digital angiography was conducted to confirm the occlusion rate. In cases of insufficient packing, an additional injection was performed using the same mixture under balloon occlusion to minimize the aneurysmal open space. By use of this approach, a maximum of two injections was required to pack the aneurysms.

### 2.5. Sampling of Aneurysms

Immediately after endovascular embolization of the aneurysms (t0) and at 7 (t7), 30 (t30), and 90 days after this surgical procedure (t90), two swine were sacrificed by anesthetic overdose (isoflurane) and submitted to surgery to remove the experimentally induced aneurysms. The experimentally induced defects were excised in toto and immediately placed into 10% buffered formalin fixative.

### 2.6. Histopathology

The formalin-fixed experimentally induced aneurysms were routinely processed for paraffin embedding. Here, 5 μm serial sections were dewaxed in xylene, rehydrated in a graded alcohol series, and stained with hematoxylin and eosin (H-E) for routine examination, Masson’s trichrome stain to evaluate connective tissue, Perls’ Prussian blue stain to detect hemosiderin deposits, and picrosirius red stain to determine the collagen types of contents. Slides were examined by two pathologists (N.F. and A.P.) in a blinded fashion.

### 2.7. Immunohistochemistry

For immunohistochemistry tissue sections were mounted on treated glass slides (Superfrost Plus; Menzel-Glaser, Braunschweig, Germany). Sections were dewaxed in xylene and rehydrated through graded alcohols. Heat-induced epitope retrieval with citrate buffer (pH 6.0) was performed in a microwave oven. Immunohistochemical labeling was carried out manually with the Sequenza slide rack and cover-plate system (Shandon, Runcorn, UK). Non-specific peroxidase activity was blocked with Bloxall blocking solution (SP-6000; Vector Laboratories, Newark, CA, USA), and aspecific antigen binding was blocked with UltraVision Protein Block (TA-060-PBQ; Thermo Scientific, Cheshire, UK). Serial sections underwent a panel of primary antibodies and were left to incubate at 4 °C overnight. Anti-Iba1 rabbit polyclonal antobody (Wako, Neuss, Germany; dilution 1:300), anti-CD3 rabbit polyclonal antibody (Dako, Glostrup, Denmark; dilution 1:200), and anti-Cd79a mouse monoclonal antibody (Exbio, Praha, Czech Republic; dilution 1:100) were used for macrophages, T-lymphocytes, and B-lymphocytes, respectively. Antibody binding was detected by the Biotinylated horse anti-Mouse/Rabbit IgG antibody (H + L) R.T.U. (Vectors Laboratories, CA, USA), the streptavidin-biotin-peroxidase kit (Vector Laboratories, Newark, CA, USA) and the 3,30-diamino-benzidine as chromogen (Vector Laboratories, Newark, CA, USA) as indicated by the manufacturer’s instructions. Stained slides were subsequently counterstained in hematoxylin followed by dehydration in graded alcohols and cleared with xylene. Sections were mounted in DPX (08600E; Surgipath Europe Ltd., Bretton, UK). The primary antibody was substituted with an unrelated matched primary antibody to serve as a negative control. Serial sections of a rat lymph node served as a positive control.

### 2.8. Morphometrical Studies

The aneurysm wall was submitted to a morphometric investigation. Bright-field images were acquired at ×40 magnification with a Leica Microsystem DFC490 digital camera mounted on a Leica DMR microscope (Wetzlar, Germany). Counting was performed using a semiautomatic analysis system (LASV 4.3, Leica) on six 15,000 μm^2^ random fields of three different areas of the aneurysm wall. These sampled areas were used to evaluate the thickness of the aneurysm wall, the thickness of the connective tissue production, the new blood vessel count, and the composition of cell populations involved in the inflammatory reaction.

### 2.9. Statistical Analysis

SPSS Advanced Statistics 21.0 statistical software (SPSS Inc., Chicago, IL, USA) was used to conduct the analysis. The ANOVA test was employed to compare the various parameters observed at different time points. Post hoc analysis was carried out using the Bonferroni test. Statistical significance was set at a 5% (alpha = 0.05) significance level.

## 3. Results

All the scheduled interventions were completed and all the swine underwent aneurysm creation and embolization. A total of 18 aneurysms were considered. In 77.78% of cases (14/18), aneurysms were successfully and completely fulfilled by the mixture without immediate or delayed complications. In 11.11% of cases (2/18), aneurysms were completely fulfilled but after balloon deflation embolic complication occurred as the mixture migrated in the parent artery. In 5.56% of cases (1/18), the rupture of the balloon catheter occurred with consequent mixture migration in healthy territory. In 5.56% of cases (1/18), the aneurysm was not treated because the swine was suppressed before the treatment. Looking at the swine, their fates were as follows: in 66.67% of cases (6/9), swine survived after the procedure and underwent scheduled controls and sacrifice according to the protocol. In 11.11% of cases (1/9), swine were successfully embolized but died 24 h after the procedure, probably due to ischemic complications. In 22.22% of cases (2/9), swine were suppressed immediately after the procedure due to embolic or hemorrhagic complications.

### 3.1. Endovascular Procedure

Upon injection, GEL first took the appearance of a thread-like structure before expanding into a huge, circular formation that occupied the aneurysm’s whole lumen. Following the GEL injection, it was possible to withdraw the balloon catheter and the microcatheter with ease. This allowed for more packing of the aneurysm by moving the microcatheter forward and injecting the mixture again while inflating the balloon catheter.

**Figure 1:**
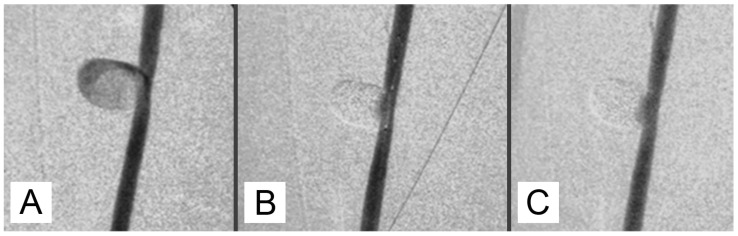
(**A**) Carotid angiography shows the aneurysm before embolization. (**B**) Attempted embolization using GEL 2:0, 5:1, via a microcatheter inserted into the microaneurysm, with balloon protection (**C**).

One possible method of cleaning a microcatheter lumen is to flush it with Lipiodol^®^. Nevertheless, it was occasionally discovered that GEL remained in the hub section of a microcatheter, making guidewire insertion challenging when the microcatheter was reinserted using a guidewire. Another microcatheter was needed in this case. In 15 of the 18 aneurysms, post-packing angiography showed nearly full aneurysm packing (Figure 1).

**Figure 2:**
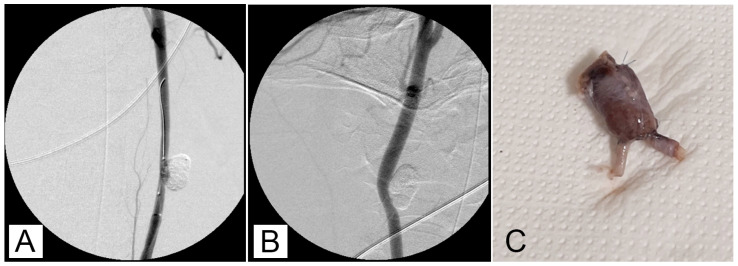
(**A**) An angiographic intraoperative control after embolization with balloon deflated. (**B**) Follow-up control at 90 days. Occlusion remains stable. (**C**) Aneurysms excised.

After 30 min of packaging, there was no migration or leakage of GEL. Post-procedure angiographic controls showed a stable occlusion of all the aneurysms with no signs of recurrence (Figure 2A,B).

### 3.2. Macroscopic Study

**Figure 3:**
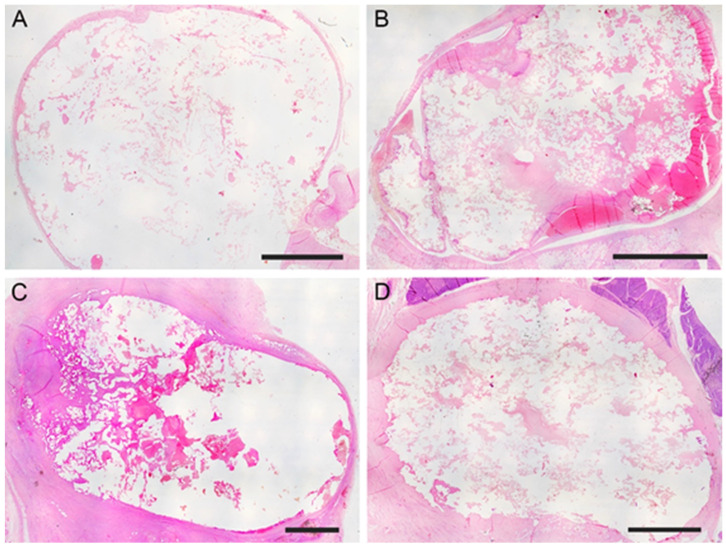
Aneurysms embolized using the modified mixture. (**A**) After the embolization, complete occlusion due to the embolizing mixture resulting as a refractile material was evident (H-E, bar = 0.5 cm). (**B**) One week post embolization, the aneurysm lumen was filled by the embolizing mixture mixed with fibrin, and small hemorrhagic areas were evident close to the wall of the structure (H-E, bar = 1 cm). (**C**) Thirty days after surgery, the aneurysm was completely occluded by the modified mixture (H-E, bar = 0.5 cm). (**D**) Ninety days after embolization, the aneurysm lumen was still occluded, and recanalization phenomena were not evident (H-E, bar = 0.5 cm).

The subjects sampled at 7, 30, and 90 days post-surgery did not exhibit symptoms related to a painful state or signs of side effects after aneurysm embolization. During the surgery for removing the experimentally induced defects, no alterations due to an ongoing inflammatory process were evidenced (Figure 2C). The mean long × short diameters of the created aneurysm ranged from 5 to 30 mm (mean diameter 12.4 mm ± 6.2 mm). The mean neck width ranged from 3 to 10 mm. The dome-to-neck ratio (long diameter/neck width) ranged from 1 to 3. A complete occlusion was observed starting from time 0 (Figure 3A) and remained evident at 7 (Figure 3B), 30 (Figure 3C), and 90 (Figure 3D) days post-surgery. The aneurysm occlusion was due to a mixture of the embolic agent and thrombotic materials. The distribution of the embolic material inside the embolic cast was homogeneous, and the embolizing material adhered to the aneurysm wall. There was no evidence of recanalization of the embolic cast in samples examined, particularly after 7, 30, and 90 days post-surgery.

### 3.3. Vascular Changes

**Figure 4:**
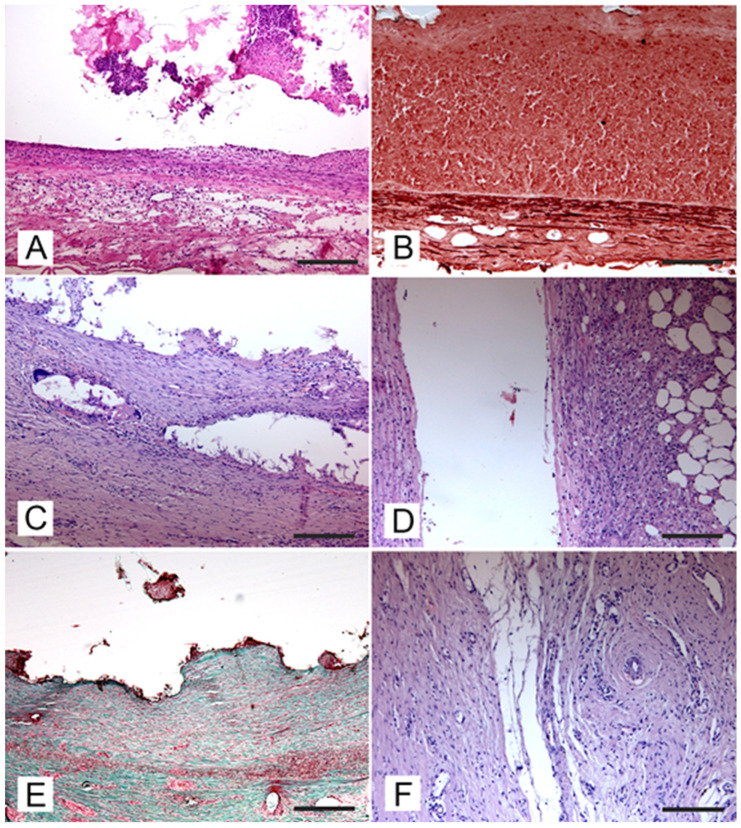
Aneurysm walls at different times after embolization: A and B, after one week, C and D, after 30 days, and E and F, after 90 days. (**A**) Aneurysm wall one week after embolization, with an acute inflammatory reaction with widespread hemorrhages and a diffuse presence of neutrophils (H-E, bar = 50 μm). (**B**) Aneurysm wall one week after embolization, with the alteration of elastic fibers in the aneurysm wall (Orcein stain, bar = 100 μm). (**C**) Aneurysm wall 30 days after embolization, with thickening of the aneurysm wall due to immature connective tissue and the presence of small glue deposits delimited by a chronic inflammatory reaction (H-E, bar = 100 μm). (**D**) Aneurysm wall 30 days after embolization, with host tissues at the periphery of experimental aneurysm showing a reduced inflammatory reaction (H-E, bar = 200 μm). (**E**) Aneurysm wall 90 days after embolization; the aneurysm wall was constituted by well-differentiated connective tissue with a reduced inflammatory reaction (Masson trichrome Goldner stain, bar = 100 μm). (**F**) Aneurysm wall 90 days after embolization, with the absence of edema and inflammatory phenomena at the periphery of the aneurysm wall (H-E, bar = 200 μm).

Vascular integrity of the aneurysm wall was observed in all experimentally induced defects, and no evidence of the perivascular extravasation of embolic material was detected in any specimens. An acute inflammatory reaction characterized by the presence of hemorrhages was detected in all samples resected after a few hours (t0) and 7 days post-surgery (Figure 4A). In samples collected after 7 days post-surgery, angionecrosis demonstrated by the alteration of elastic fibers was also observed (Figure 4B). At 30 days post-surgery, the aneurysm wall was slightly thickened due to the proliferation of a poorly differentiated connective tissue and the presence of chronic vascular inflammation (Figure 4C). In the aneurysm wall, scattered foreign body reactions at the periphery of embolizing material residues characterized by the presence of foreign body giant cells were also detected (Figure 4D). At the periphery of the aneurysm wall, there was edema and host tissue which showed reduced inflammatory infiltration (Figure 4E). Ninety days post-surgery, the aneurysm wall was thickened and consisted of well-differentiated connective tissue (Figure 4F). Pigment deposits of hemoglobin origin (both hemosiderin and hematin) were also observed.

### 3.4. Perivascular Changes

In samples collected 7 days post-surgery, a limited perivascular edema was observed. Thirty days post-surgery, a marked reduction in the perivascular edema was evident, and scattered perivascular inflammatory infiltrates constituted by macrophages and small lymphocytes were also present. No perivascular changes were detected in samples collected 90 days post-surgery.

### 3.5. Immunohistochemical Studies

Immunohistochemical studies revealed the presence of a reduced number of T and B lymphocytes in the inflammatory infiltrates observed in the aneurysm wall 7 and 30 days post-surgery. Many macrophages were detected in chronic vascular inflammatory infiltrates detected in the aneurysm wall 30 days post-surgery. The number of macrophages in the aneurysm wall was drastically reduced in samples collected 90 days post-surgery.

### 3.6. Morphometric Analysis

**Figure 5:**
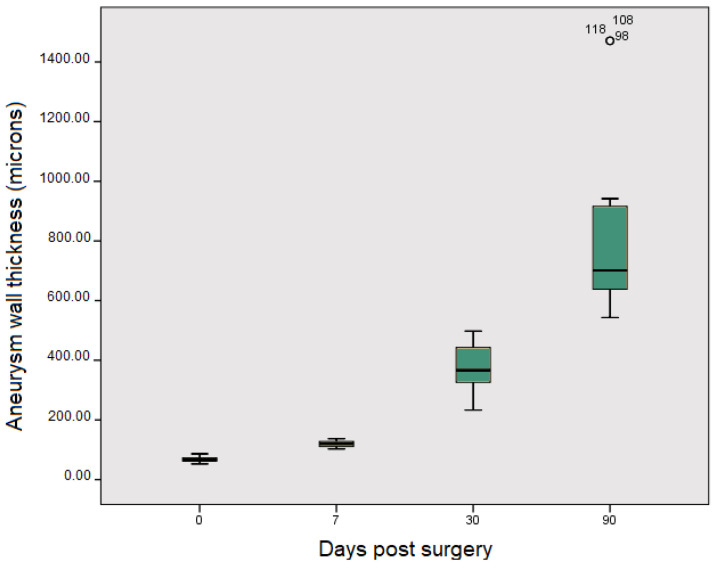
Aneurysm wall thickness at different times after embolization.

The results of morphometric analysis are presented in Figure 5.

Morphometric analysis performed to determine the thickness of the aneurysm wall at different times from embolization revealed that at 7 days post embolization, the aneurysm walls were only slightly increased due to the acute inflammatory reaction to the mixture deposit without significant differences (65 μm ± 9 μm at t0 vs. 120 μm ± 10 μm at t7). Thirty days post embolization, there was a significant increase in the aneurysm wall thickening (120 μm ± 10 μm al t7 vs. 366 μm ± 83 μm at t30; *p* < 0.001) due to the connective tissue proliferation, which was more evident 90 days after embolization (366 μm ± 83 μm al t30 vs. 701 μm ± 261 μm at t90; *p* < 0.001).

## 4. Discussion

This is the first experimental study to address the feasibility, efficacy, and tissue reaction, after embolization with a mixture of n-butyl-cyanoacrylate (Glubran 2^®^), ethanol, and lipidol^®^, of aneurysms experimentally induced in swine. The endovascular treatment of cerebral aneurysms has become an alternative to conventional neurosurgical clipping [27]. Guglielmi Detachable Coils (GDCs) offer safe and effective endovascular treatment for cerebral aneurysms [28] but face limitations in dense packing and control due to their ineffectiveness in wide-necked or large aneurysms, making them less effective [29,30,31,32,33]. One potential alternative for the treatment of certain cerebral aneurysms is the use of liquid embolic agents [34,35]. However, these treatment approaches have not gained widespread acceptance due to inherent technical constraints. One significant drawback has been the inability to adjust or retrieve the liquid embolic agent once delivered into the aneurysm sac [36]. Several clinical and experimental techniques, including the placement of metallic stents, balloon inflation across the aneurysm’s neck, and intra-aneurysmal flow control with proximal balloon protection, have been used to reduce the liquid embolic agent’s distal migration [34,37]. Kawai et al. successfully packed a narrow-neck carotid artery aneurysm with NLE, but not wide-neck aneurysms due to the NLE migration risk [15]. The studies by Tanaka et al. [38] and Hama et al. [39] represent a step forward, as balloon inflation at the aneurysm site was effectively used to execute the NLE packing of aneurysms with a neck width of ≥6.3 mm and a dome-to-neck ratio of <2.0. The balloon-assisted injection of liquid embolic agents and balloon- or stent-assisted coiling (BAC and SAC) use a two-catheter system, requiring glue/dimethyl sulfoxide-compatible catheters and balloons, and the aneurysm sac is embolized with liquid agents. Similar occlusion rates were seen at in the CAMEO trial [40], which examined the treatment of 100 aneurysms using the Onyx liquid embolic system. Using this system, a 79% complete occlusion rate at 12 months was obtained, but the rate of major adverse events (26.8%) was remarkable. This procedure is still used in other nations, but it did not catch on in the United States due to the high rate of complications.

In this study, seven days after the embolization, a mild acute vascular reaction was evident in the aneurysm wall, probably related to the polymerization of the cyanoacrylic glue present in the mixture, without the involvement of host tissues at the periphery of the experimentally induced lesion. This acute reaction was followed by chronic inflammation which induced a thickening of the aneurysm wall due to the proliferation of connective tissue still confined within the aneurysm wall. The embolization procedure induced vascular changes followed by a complete and stable occlusion of the aneurysms. Our experimental study demonstrated that the presence of the occlusion remained stable up to 90 days after surgery, without recanalization phenomena.

The 2:0, 5:1 ratios of GEL components seemed to offer a good compromise between polymerization time, balloon resistance, and catheter patency. As is well known, the Glubran^®^ + Lipiodol^®^ combination is not suitable for packing wide-neck aneurysms [41] due to the formation of tiny, oily droplets and its strong adhesion to the balloon catheter and micro-catheter [42], which may make it impossible to remove from the vessel lumen. GEL, on the other hand, produced a single huge solid droplet, which made it look like a suitable material for aneurysm packing [38,39,40,41,42,43]. GEL was less adherent to the balloon catheter and microcatheter than G + L was. This allowed the microcatheter to be advanced further into the aneurysm and GEL to be reinjected for more packing via the microcatheter. The GEL’s minimal stickiness made it simple to remove both catheters. In two of the eighteen aneurysms, we saw resistance to the microcatheter during retrieval, despite the fact that the microcatheter did not adhere to the adhesive cast.

The GEL mixture in our study exhibited outstanding visibility under fluoroscopy. To prevent leakage into the parent artery and distal migration of the adhesive, liquid embolic agents should be employed under high-quality, subtracted, real-time fluoroscopic guidance in addition to having optimal visibility. During the aneurysm packing, the continuous injection of GEL was necessary to prevent blood regurgitation. Ethanol accelerates polymerization along the GEL mixture–blood interface, potentially reducing unwanted NBCA adhesion to the catheter by binding to anions in the blood [30]. Since the hemodynamics of a human aneurysm differ from those of the surgically created side-wall aneurysm model in pigs, the direct application of our findings to clinical research may be limited. Furthermore, following very long-term follow-up, the embolic effect’s stability and safety must be verified. Taking everything to account, the glue embolization of aneurysms is technically feasible with neck protection of the flow with a balloon.

In conclusion, the GEL mixture may be useful for embolizing arteriovenous malformations, controlling acute bleeding, and isolating aneurysms in future clinical applications, requiring balloon occlusion and selective catheterization. GEL could also shorten the treatment time according to its very short polymerization time and could be used in combination with detachable coils for aneurysm packing. In short, despite certain restrictions, packing for aneurysms using GEL is safe and feasible, with minimal adhesion to the microcatheter and embolic complications.

## Data Availability

The raw data supporting the conclusions of this article will be made available by the authors on request.
